# Risk of Subsequent Knee Arthroplasty After Sports Medicine Procedures

**DOI:** 10.5435/JAAOSGlobal-D-20-00125

**Published:** 2020-08-10

**Authors:** Lawrence C. Enweze, Kunal Varshneya, Seth L. Sherman, Marc R. Safran, Geoffrey D. Abrams

**Affiliations:** From the Department of Orthopaedic Surgery, Stanford University School of Medicine, Stanford, CA.

## Abstract

**Introduction:**

Approximately 10% of men and 13% of women older than the age of 60 are affected by symptomatic osteoarthritis of the knee. Anatomic repair or reconstruction after knee injury has been a central tenet of surgical treatment to reduce the risk of osteoarthritis. The purpose of this study was to examine common sports medicine procedures of the knee and determine the proportion of patients who subsequently undergo total knee arthroplasty (TKA).

**Methods:**

The MarketScan database was queried from the period of January 2007 through December 2016. Patients were identified, who underwent a procedure of the knee, as defined by Current Procedural Terminology codes relating to nonarthroplasty procedures of the knee. Patients in whom laterality could not be confirmed or underwent another ipsilateral knee procedure before TKA were excluded from this study. The primary outcome of this study was the overall rate of TKA after index knee surgery. Time from index procedure to TKA was a secondary outcome. A multivariate regression analysis was used to control for covariates such as age, sex, and comorbidity status.

**Results:**

A total of 843,749 patients underwent one of the 13 common sports medicine procedures of the knee. The procedure with the highest unadjusted rate of subsequent TKA was arthroscopic osteochondral allograft (5.81%), whereas anterior cruciate ligament (ACL) reconstruction with meniscus repair demonstrated the lowest rate of subsequent TKA (0.01%). When adjusting for confounding factors, the regression analysis identified meniscal transplantation (odds ratio [OR] = 3.06, *P* < 0.0001) as having the highest risk of subsequent TKA, followed by osteochondral autograft (OR = 1.74, *P* = 0.0424) and arthroscopic osteochondral allograft (OR = 1.49, *P* < 0.0001). ACL reconstruction with meniscus repair (OR = 0.02, *P* < 0.0001), ACL reconstruction alone (OR = 0.17, *P* < 0.0001), ACL with meniscectomy (OR = 0.20, *P* < 0.0001), and meniscal repair (OR = 0.65, *P* < 0.0001) had the lowest rate of subsequent TKA. ACL reconstruction with meniscus repair had the longest period from index procedure to TKA at 2827 days.

**Conclusion:**

ACL reconstruction and meniscus preservation demonstrated an extremely low rate of conversion to TKA when compared with patients who needed salvage interventions such as meniscus and cartilage transplantation. None of the salvage interventions delayed the need for a TKA. Meniscal transplantation had the highest risk of all procedures of going on to a TKA.

In the United States, it is estimated that 10% of men and 13% of women older than the age of 60 are affected by symptomatic osteoarthritis (OA) of the knee.^1^ The incidence of OA will likely continue to increase because of an aging population and prevalence of obesity.^1^ The initiation of arthritis is most commonly because of mechanical insult, leading to damage of the intra-articular structures of the knee, such as the hyaline cartilage or menisci.^2,3^ In some patients, these events result in a continual low-grade inflammatory degenerative process within the joint, which over many years may lead to frank arthritis. Owing to the limited or avascular nature of the menisci and hyaline cartilage, these structures have very poor regenerative potential. Damage to these structures leads to increased force on the remaining cartilage in the joint, resulting in further damage.^4^

Anatomic repair or reconstruction of native knee anatomy is a central tenet of sports medicine. Restoring or preserving the normal anatomy of the knee leads to restoration of function but can also return knee biomechanics to the intact state.^5,6,7,8,9^ This in turn can reduce loading and sheer forces on the hyaline cartilage.^10,11^ Many surgeries were done that seek to restore the normal structure and function of the knee. In particular, there has been an increased emphasis on meniscal preservation and anatomic anterior cruciate ligament (ACL) reconstruction.^5,12,13^ More recently, cartilage and meniscus restoration procedures—such as autologous chondrocyte implantation (ACI), osteochondral autograft, and allograft—and meniscus transplantation procedures have become more common. These procedures are typically done in more complex knee pathologies, particularly when previous surgery has failed. Some practitioners state that these procedures can reduce the risk of future arthritis, but limited data exist to support such a claim.^14,15,16,17,18^

For patients with knee pain, procedures are available that can help relieve symptoms. Meniscectomies and débridement for degenerative meniscal tears, along with chondroplasty, synovectomy, and bone marrow stimulating procedures are thought of as procedures for more short-term relief of knee symptoms.^19^ Controversy exists regarding the efficacy of these procedures. Some studies have showed no benefit in functional outcome scores for these types of procedures when compared with sham surgery or physical therapy,^20,21^ whereas others have shown some benefit even up to 10 years postoperatively.^22-24^ Little data exist regarding the mean period between joint preserving procedures and total knee arthroplasty (TKA).

The purpose of this study was to examine 13 nonarthroplasty procedures of the knee to determine the proportion of patients who subsequently underwent TKA. We hypothesized that patients undergoing ACL reconstruction and meniscus repairs, after controlling for age, sex, and comorbidities, would have a lower risk of subsequent TKA versus those undergoing salvage procedures such as meniscus transplantation and osteochondral allograft.

## Methods

### Data Source

This study obtained a sample of the MarketScan Commercial Claims and Encounters database (Truven Health Analytics, Ann Arbor, MI) from January 1, 2007, to December 31, 2016. This is a national database with a collection of commercial inpatient, outpatient, and pharmaceutical claims of more than 75 million employees, retirees, and dependents representing a substantial portion of the US population covered by employer-sponsored insurance. MarketScan contains 53 million patients' inpatient records, 40 million with employer-sponsored insurance, 3.7 million with Medicare Part B, and 6.8 million on Medicaid for a total of over 28 billion patient records. MarketScan databases are built from privately insured medical claims by employers who have established contracts with Truven Health. Approximately 350 insurance payers exist in this data set. Truven Health maintains a unique person-level identifier for each individual and follows them in a longitudinal manner. The person-level identifier is consistent throughout the data set. Owing to MarketScan's sourcing from large employers, its data boast superior longitudinal tracking of patients. MarketScan is fully Health Insurance Portability and Accountability Act compliant and undergoes periodic reviewal to ensure the integrity of the data. All data from these databases are de-identified, and thus, these data and study are exempt from institutional review board approval in accordance with the Health Insurance Portability and Accountability Act of 1996.^25^ For this study, primarily the outpatient services data were used. The MarketScan database contains International Classification of Diseases, Ninth Revision, Clinical Modification and 10th revision, Clinical Modification, Current Procedural Terminology (CPT), Diagnosis Related Group codes and National Drug Codes.

### Inclusion Criteria

Records were queried to identify patients who underwent a primary knee surgery, defined in this study by the presence of a CPT code for the following non-TKA knee procedures: synovectomy (CPT 29875 or 29876), chondroplasty (CPT 29877), meniscus débridement (CPT 29880 or 29881), microfracture (29879), meniscus repair (CPT 29882 or 29883), ACL reconstruction alone, ACL with meniscectomy, ACL with meniscus repair, (CPT 29888 with or without 29880, 29881, 29882, or 29883 for concomitant meniscus procedure), meniscus transplant (29868), osteochondroplasty allograft, open (CPT 27415) osteochondroplasty, autograft (29866) osteochondroplasty, allograft, scope (29867), or ACI (CPT 27412). The index procedure (first knee surgery in the outpatient setting) was used for the analysis. Patients without laterality indication, undergoing revision procedures, or who had another ipsilateral knee procedure between index procedure and TKA were excluded. Patient level data, such as age, sex, and comorbidity status were also determined. In this study, comorbidity status was measured by calculating the Charlson Comorbidity Index (CCI).

### Outcomes

The primary outcome of this study was the overall rate of TKA after index knee surgery. The records of MarketScan data were queried to find the index TKA procedure for patients who underwent one of the aforementioned knee surgeries. Patients who had the CPT code for TKA (27446 or 27447) on the ipsilateral knee after the primary knee surgery were categorized as subsequent TKA patients. Days to subsequent knee surgery (TKA and non-TKA) were determined by finding the difference between the date of the index nonarthroplasty surgery and index TKA.

### Analyses

Frequencies of patients undergoing knee surgeries were tabulated, and proportions requiring TKA procedures were calculated. A multivariate regression analysis was conducted to control for covariates such as age, sex, and comorbidity status (in the form of CCI). An odds ratio (OR) was calculated to determine the risk of a patient who underwent each specific index procedure of progressing to a TKA when compared with the entire cohort, after controlling for, age, sex, and CCI. Two sample Student *t*-test, chi-squared tests, or Fisher exact tests were used as appropriate. An alpha level of 0.05 was used to discern statistical significance. The cohort was built using the cloud-based tool Redivis (Redivis), and statistical analysis was conducted in SAS 9.4 (SAS Institute).

## Results

Between 2007 and 2016, a total of 843,749 subjects underwent 13 index procedures who were chosen for this study (Table 1). Meniscal débridement accounted for most operations done in this cohort. Some of the least common procedures were meniscal transplant (0.06%), ACI (0.08%), osteochondral autograft (0.13%), and open osteochondral allograft (0.18%). Isolated ACL reconstruction (28.8), ACL + meniscus repair (24.6), and meniscal repair (30.1), all had a mean age of less than or equal to 30 years of age. Procedures that had subjects with a mean age younger than 40 years were meniscus débridement, microfracture, arthroscopic osteochondral allograft (44.2), chondroplasty (42.8), and synovectomy (40.79).

**Table 1:** Baseline Demographics of nonTKA Index Procedures

Procedure	N	%	Age, Mean	Age, SD	Age, Range	Female Sex, %	CCI, Mean
ACI	696	0.08	30.2	10.3	12-61	45.6	0.004
ACL + meniscectomy	44,421	5.26	31.1	13	3-64	38.2	0.009
ACL + meniscus repair	13,327	1.58	24.6	10.5	5-64	42.5	0.003
Chondroplasty	78,120	9.26	42.8	13.5	14-65	57.3	0.028
Isolated ACL	87,998	10.43	28.8	12.2	6-65	45.6	0.005
Meniscus débridement	565,959	67.08	48.9	11.7	13-65	45.7	0.033
Meniscus repair	17,341	2.06	30.1	14.7	4-65	39.5	0.013
Meniscus transplant	537	0.06	27	10.5	8-65	49.5	0.011
Microfracture	58,076	6.88	44.6	13.1	7-65	49.1	0.031
Osteochondroplasty, allograft, open	1490	0.18	31.3	12	9-65	40.3	0.011
Osteochondroplasty, allograft, scope	45,271	5.37	44.2	14.1	4-65	51.6	0.033
Osteochondroplasty, autograft	1062	0.13	31.2	13.1	10-65	41.7	0.022
Synovectomy	79,445	9.42	40.79	15.3	5-65	53.8	0.029

ACI = autologous chondrocyte implantation, ACL = anterior cruciate ligament, CCI = Charlson Comorbidity Index, TKA = total knee arthroplasty

Of the 843,749 index procedures that were done, 23,127 of the subjects subsequently had a TKA (2.33%) (Table 2). The procedure that had the highest rate of subsequent TKA was the arthroscopic osteochondral allograft (5.81%) (Table 2). There were a variety of mean days to TKA among the various procedures. The shortest time between index procedure and TKA was 61 days. The longest time between the 2 was 3560 days (10 years). ACL + meniscus repair demonstrated the longest latency period (time between index procedure and TKA) at a mean of 2827 days (Table 2). For meniscal sacrificing and cartilage salvage procedures, the mean time to TKA ranged from 572 to 975 days.

**Table 2:** Patients Requiring Subsequent TKA on The Same Knee

Procedure	N	%	Means Days to TKA	SD	Range
ACI	3	0.43	652.3	54.1	602-711
ACL + meniscectomy	69	0.16	777.2	500.7	78-2574
ACL + meniscus repair	1	0.01	2827.0	n/a	n/a
Chondroplasty	2048	2.62	572.0	401	101-3415
Isolated ACL	93	0.11	956.0	738.7	108-3181
Meniscus débridement	16524	2.92	604.0	563.7	61-3560
Meniscus repair	103	0.59	608.5	525.3	90-2846
Meniscus transplant	8	1.49	975.9	583.1	399-1980
Microfracture	1662	2.86	561.6	480	62-3143
Osteochondroplasty, allograft, open	18	1.21	653.2	416	180-1637
Osteochondroplasty, allograft, scope	1102	5.81	577.9	402.8	111-3507
Osteochondroplasty, autograft	14	0.07	627.1	344.8	78-1115
Synovectomy	1482	1.87	579.3	468.5	65-3507

ACI = autologous chondrocyte implantation, ACL = anterior cruciate ligament, TKA = total knee arthroplasty

When adjusting for age, sex and CCI, meniscal transplantation (OR = 3.06, *P* < 0.0001) had the highest risk of subsequent TKA (Table 3). This was followed by osteochondral autograft (OR = 1.74, *P* = 0.0424), arthroscopic osteochondral allograft (OR = 1.49, *P* < 0.0001), chondroplasty (OR = 1.43, *P* < 0.0001), microfracture (OR = 1.39, *P* < 0.0001), and meniscal débridement (OR = 1.25, *P* < 0.0001). ACL + meniscus repair (OR = 0.02, *P* < 0.0001), isolated ACL reconstruction (OR = 0.17, *P* < 0.0001), ACL + meniscectomy (OR = 0.2, *P* < 0.0001), and meniscal repair (OR = 0.65, *P* < 0.0001) had the lowest risk of future TKA after controlling for differences between groups (Table 3).

**Table 3:** Risk for Subsequent TKA

Variable	Adjusted ORs		
	OR	95% CI	*P*
Meniscus transplant	3.06	1.38-5.83	<0.0001
Osteochondroplasty, autograft	1.74	0.97-2.86	0.0424
Osteochondroplasty, allograft, scope	1.49	1.32-1.67	<0.0001
Osteochondroplasty, allograft, open	1.48	0.89-2.31	0.1013
Chondroplasty	1.43	1.35-1.51	<0.0001
Microfracture	1.39	1.31-1.47	<0.0001
Meniscus débridement	1.25	1.19-1.32	<0.0001
Synovectomy	0.72	0.65-0.80	<0.0001
Meniscus repair	0.65	0.54-0.79	<0.0001
ACI	0.58	0.14-1.51	0.3517
ACL reconstruction + meniscectomy	0.20	0.16-0.26	<0.0001
Isolated ACL reconstruction	0.17	0.13-0.20	<0.0001
ACL reconstruction + meniscus repair	0.02	0.00-0.09	<0.0001

ACI = autologous chondrocyte implantation, ACL = anterior cruciate ligament, CI = confidence interval, OR = odds ratio, TKA = total knee arthroplasty

## Discussion

We found that the overall rate of subsequent TKA after an index nonarthroplasty procedure was low at just over 2%. The highest risk for TKA conversion was for osteochondral allograft and meniscus transplant procedures. The rates of TKA, even in these salvage procedures, remained low however. The lowest rate of subsequent TKA was for ACL reconstruction with meniscus repair. Procedures such as microfracture, meniscectomy, and synovectomy demonstrated the lowest average number of days between index procedure and TKA. These data highlight the importance of preservation and restoration of native anatomy and biomechanics of the knee through reconstruction and repair to prevent early OA. These data also highlight that of the débridement or cartilage/meniscus reconstructive procedures (synovectomy, chondroplasty, meniscectomy, osteochondral allograft and autograft, and meniscal transplantation), no single procedure reduced the risk of subsequently undergoing a TKA. Indication and choice of salvage/palliative procedure should be based on clinical symptoms and not the desire to delay the need for a TKA.^26-28^

For those procedures with a low risk of subsequent TKA, the ACL reconstruction with the meniscus repair group was the most protective (OR = 0.02). This supports previous findings that reconstruction of the ACL and preservation of meniscus tissue is important in the reduction and/or prevention of cartilage damage.^10,11^ The importance of meniscus repair is in keeping with past literature.^8,9^ Johnson et al found that at almost 11 years after isolated meniscus repair, minimal joint changes were seen in only 8% of his patients on the surgical knee with 3% of patients having changes also on the contralateral side. He evaluated radiographs and found joint changes in 69% of his patients after a meniscectomy was done.^8,29^ Sommerlath et al also found better functional outcomes in patients with meniscal repairs versus meniscectomy.^30^ The meniscus is an important structure because it acts to increase the congruency of the tibiofemoral joint to help transmit load seen in the joint and decreases the stress on the articular cartilage.^31^ It also helps to preserve the cartilage by providing rotational stability and lubrication and nutrition to the articular cartilage.^32,33^

Consistent with the importance of the meniscus in joint preservation, we found that among ACL patients, those undergoing meniscectomy in conjunction with ACL reconstruction had the greatest risk of subsequent TKA. This was followed, in order of decreasing risk of TKA, by isolated ACL reconstruction and ACL reconstruction with meniscus repair. Furthermore, a recent investigation reported a 3.62 relative risk of OA of the knee after an ACL reconstruction knee when compared with the uninjured knee.^5^ A 4.89 relative risk, however, was seen with ACL deficient knees treated without reconstruction. The study's conclusion was that an ACL injury increases the risk for OA, but ACL reconstruction can slightly decrease the risk of going on to OA when compared with conservative treatment.^5^

Meniscus transplantation had the greatest risk for subsequent TKA in our study population, with nearly 1.5% of patients continuing on to TKA. Given that these procedures are often done in salvage and revision cases, often with cartilage degeneration, the rate of TKA conversion over the study timeframe is relatively low. Meniscal transplantations were first done in 1984 and since then, the chondroprotective nature of the procedure has always been debated.^34^ In theory, preservation of meniscal tissue through a cadaveric allograft would have the same chondroprotective benefits as native meniscal tissue. Recent systematic reviews have investigated whether the procedure may play a role in decreasing the progression of OA.^35,36^ These investigations concluded that there may be evidence that it may protect against future cartilage damage but does not stop the overall degenerative process of healthy cartilage. Furthermore, meniscus transplantation is a technically demanding procedure, with restoration of native knee biomechanics closely related to proper surgical technique in allograft sizing, root placement, and meniscus attachment.^37-39^

Other than meniscus transplant, procedures that had the highest mean age at index procedure also had the highest rate of subsequent TKA—arthroscopic osteochondral allograft, meniscal débridement, microfracture, chondroplasty, and synovectomy. Apart from osteochondral allograft procedure, most of these are procedures that are often used as palliative procedures for patients with some degenerative change of the knee who have failed conservative treatment. In this study, these five procedures also had the lowest average number of days before receiving a subsequent TKA (range: 561-604 days). This suggests the likelihood that many of these procedures do not alter the natural history of OA of the knee or the eventual need for a TKA.

When controlling for age, sex, and comorbidities, the only procedure among those with the oldest average age to have a negative correlation with subsequent TKA was the synovectomy group (OR = 0.72). This may be for a multitude of reasons. Of the procedures, this is the only procedure that does not deal with the meniscus or cartilage pathology, so it is conceivable that patients in this cohort had a milder disease. However, synovectomy had a similar OR to the meniscal repair group (OR = 0.65). It is possible that synovectomy might have a similar effect. It is now also known that OA likely is caused by low grade inflammation.^32,40^ OA may be propagated by inflammatory mediators released by cartilage, subchondral bone, and the synovium.^32,40,41^ A synovectomy may play a vital role in dampening the inflammatory response in the knee joint and delaying the degenerative process. More studies should be done looking at procedures specifically addressing inflammation around the cartilage to determine whether it has the similar protective effects.

As with all investigation, limitations exist. The results of this study are dependent on the data included in the database, and this leads to the possibility that not all patients who underwent TKA are captured in the database. This is also a retrospective database study, and it could not qualitatively measure the extent of degeneration or injury of the knee at the time of the index procedure and at the time of the arthroplasty procedure. It also could not assess the surgeon's technical skills, surgeon volume, surgical techniques, graft type, cartilage graft viability, transplantation techniques, amount of OA or malalignment, any iatrogenic injury during the initial procedure, or patient's adherence to postoperative protocols, activity limitations, or concomitant knee diagnosis that may not have been diagnosed or warrant a procedure. In addition, in this study, TKA is considered the end time point and represents, in theory, severe OA. However, many factors exist that may cofound who gets a TKA and when. This is an elective procedure, and factors such as patient preference, surgeon's indication, and insurance status cannot be controlled for.

## Conclusions

ACL reconstruction and meniscus preservation demonstrated an extremely low rate of conversion to TKA when compared with patients who needed salvage interventions such as meniscus and cartilage transplantation. Although the rates for salvage procedures such as meniscus and cartilage transplantation were increased versus other nonarthroplasty procedures, the overall rate of subsequent TKA conversion remained low.

## References

[1] Zhang Y Jordan JM: Epidemiology of osteoarthritis. Clin Geriatr Med 2010;26:355-369. 20699159 10.1016/j.cger.2010.03.001 PMC2920533

[2] Sohn DH Sokolove J Sharpe O: Plasma proteins present in osteoarthritic synovial fluid can stimulate cytokine production via Toll-like receptor 4. Arthritis Res Ther 2012;14:R7. 22225630 10.1186/ar3555 PMC3392793

[3] Safran MR Seiber K: The evidence for surgical repair of articular cartilage in the knee. J Am Acad Orthop Surg 2010;18:259-266. 20435876 10.5435/00124635-201005000-00002

[4] Sophia Fox AJ Bedi A Rodeo SA: The basic science of articular cartilage: Structure, composition, and function. Sports Health 2009;1:461-468. 23015907 10.1177/1941738109350438 PMC3445147

[5] Ajuied A Wong F Smith C: Anterior cruciate ligament injury and radiologic progression of knee osteoarthritis: A systematic review and meta-analysis. Am J Sports Med 2014;42:2242-2252. 24214929 10.1177/0363546513508376

[6] Risberg MA Oiestad BE Gunderson R: Changes in knee osteoarthritis, symptoms, and function after anterior cruciate ligament reconstruction: A 20-year prospective follow-up study. Am J Sports Med 2016;44:1215-1224. 26912282 10.1177/0363546515626539

[7] Faucett SC Geisler BP Chahla J: Meniscus root repair vs meniscectomy or nonoperative management to prevent knee osteoarthritis after medial meniscus root tears: Clinical and economic effectiveness. Am J Sports Med 2019;47:762-769. 29517925 10.1177/0363546518755754

[8] Lee WQ Gan JZW Lie DTT: Save the meniscus—Clinical outcomes of meniscectomy versus meniscal repair. J Orthop Surg (Hong Kong) 2019;27:230949901984981. 10.1177/2309499019849813 31117923

[9] Weber J Koch M Angele P Zellner J: The role of meniscal repair for prevention of early onset of osteoarthritis. J Exp Orthop 2018;5:10. 29607459 10.1186/s40634-018-0122-z PMC5879034

[10] Chu CR Williams AA West RV: Quantitative magnetic eesonance imaging UTE-T2* mapping of cartilage and meniscus healing after anatomic anterior cruciate ligament reconstruction. Am J Sports Med 2014;42:1847-1856. 24812196 10.1177/0363546514532227 PMC5278879

[11] Titchenal MR Williams AA Chehab EF: Cartilage subsurface changes to magnetic resonance imaging UTE-T2* 2 years after anterior cruciate ligament reconstruction correlate with walking mechanics associated with knee osteoarthritis. Am J Sports Med 2018;46:565-572. 29293364 10.1177/0363546517743969 PMC6548433

[12] Abrams GD Frank RM Gupta AK Harris JD McCormick FM Cole BJ: Trends in meniscus repair and meniscectomy in the United States, 2005-2011. Am J Sports Med 2013;41:2333-2339. 23863849 10.1177/0363546513495641

[13] Tibor L Chan PH Funahashi TT Wyatt R Maletis GB Inacio MCS: Surgical technique trends in primary ACL reconstruction from 2007 to 2014. J Bone Joint Surg 2016;98:1079-1089. 27385681 10.2106/JBJS.15.00881

[14] Sommerfeldt M Goodine T Raheem A Whittaker J Otto D: Relationship between time to ACL reconstruction and presence of adverse changes in the knee at the time of reconstruction. Orthop J Sports Med 2018;6:2325967118813917. 30560143 10.1177/2325967118813917 PMC6293370

[15] Cheung EC Dilallo M Feeley BT Lansdown DA: Osteoarthritis and ACL reconstruction—Myths and risks. Curr Rev Musculoskelet Med 2020;13:115-122. 31894466 10.1007/s12178-019-09596-w PMC7083996

[16] Lee BS Bin SI Kim JM: Articular cartilage degenerates after subtotal/total lateral meniscectomy but radiographic arthrosis progression is reduced after meniscal transplantation. Am J Sports Med 2016;44:159-165. 26582798 10.1177/0363546515612076

[17] Smith NA Parkinson B Hutchinson CE Costa ML Spalding T: Is meniscal allograft transplantation chondroprotective? A systematic review of radiological outcomes. Knee Surg Sports Traumatol Arthrosc 2016;24:2923-2935. 25786823 10.1007/s00167-015-3573-0

[18] Young J Tudor F Mahmoud A Myers P: Meniscal transplantation: Procedures, outcomes, and rehabilitation. Orthop Res Rev 2017;9:35-43. 30774475 10.2147/ORR.S94378 PMC6209369

[19] Khan M Khanna V Adili A Ayeni OR Bedi A Bhandari M: Knee osteoarthritis—When arthroscopy can help. Pol Arch Intern Med 2018;128:121-125. 29355845 10.20452/pamw.4186

[20] Moseley JB O'Malley K Petersen NJ: A controlled trial of arthroscopic surgery for osteoarthritis of the knee. N Engl J Med 2002;347:81-88. 12110735 10.1056/NEJMoa013259

[21] Kirkley A Birmingham TB Litchfield RB: A randomized trial of arthroscopic surgery for osteoarthritis of the knee. N Engl J Med 2008;359:1097-1107. 18784099 10.1056/NEJMoa0708333

[22] Giri S Santosha Singh CAK: Role of arthroscopy in the treatment of osteoarthritis of knee. J Clin Diagn Res 2015;9:RC08-RC11. 10.7860/JCDR/2015/13809.6390 PMC4576602 26436009

[23] Steadman JR Briggs KK Matheny LM Ellis HB: Ten-year survivorship after knee arthroscopy in patients with Kellgren-Lawrence grade 3 and grade 4 osteoarthritis of the knee. Arthroscopy 2013;29:220-225. 23273893 10.1016/j.arthro.2012.08.018

[24] Su X Li C Liao W: Comparison of arthroscopic and conservative treatments for knee osteoarthritis: A 5-year retrospective comparative study. Arthroscopy 2018;34:652-659. 29229416 10.1016/j.arthro.2017.09.023

[25] IBM Corp: Marketscan research data [online]. 2020 Marketscan.truvenhealth.com. https://marketscan.truvenhealth.com/marketscanportal/. Accessed May 31, 2020.

[26] Noyes FR Barber-Westin SD: Arthroscopic repair of meniscal tears extending into the avascular zone in patients younger than twenty years of age. Am J Sports Med 2002;30:589-600. 12130415 10.1177/03635465020300042001

[27] Krych AJ McIntosh AL Voll AE Stuart MJ Dahm DL: Arthroscopic repair of isolated meniscal tears in patients 18 years and younger. Am J Sports Med 2008;36:1283-1289. 18319351 10.1177/0363546508314411

[28] Eggli S Wegmüller H Kosina J Huckell C Jakob RP: Long-term results of arthroscopic meniscal repair: An analysis of isolated tears. Am J Sports Med 1995;23:715-720. 8600740 10.1177/036354659502300614

[29] Sommerlath KG: Results of meniscal repair and partial meniscectomy in stable knees. Int Orthop 1991;15:347-350. 1809716 10.1007/BF00186875

[30] Fox AJS Wanivenhaus F Burge AJ Warren RF Rodeo SA: The human meniscus: A review of anatomy, function, injury, and advances in treatment. Clin Anat 2015;28:269-287. 25125315 10.1002/ca.22456

[31] Makris EA Hadidi P Athanasiou KA: The knee meniscus: Structure–function, pathophysiology, current repair techniques, and prospects for regeneration. Biomaterials 2011;32:7411-7431. 21764438 10.1016/j.biomaterials.2011.06.037 PMC3161498

[32] Stanford University, Stanford, CA: Meniscal damage, partial medial meniscectomy, and the development of osteoarthritis. https://purl.stanford.edu/rr943bf5062. Accessed September 2, 2019.

[33] Berenbaum F: Osteoarthritis as an inflammatory disease (osteoarthritis is not osteoarthrosis!). Osteoarthritis Cartilage 2013;21:16-21. 23194896 10.1016/j.joca.2012.11.012

[34] Smith NA Parkinson B Hutchinson CE Costa ML Spalding T: Is meniscal allograft transplantation chondroprotective? A systematic review of radiological outcomes. Knee Surg Sports Traumatol Arthrosc 2016;24:2923-2935. 25786823 10.1007/s00167-015-3573-0

[35] Samitier G Alentorn-Geli E Taylor DC: Meniscal allograft transplantation. Part 2: Systematic review of transplant timing, outcomes, return to competition, associated procedures, and prevention of osteoarthritis. Knee Surg Sports Traumatol Arthrosc 2015;23:323-333. 25266230 10.1007/s00167-014-3344-3

[36] Lee DW Park JH Chung KS Ha JK Kim JG: Arthroscopic medial meniscal allograft transplantation with modified bone plug technique. Arthrosc Tech 2017;6:e1437-e1442. 29354454 10.1016/j.eats.2017.06.007 PMC5622413

[37] Stevenson C Mahmoud A Tudor F Myers P: Meniscal allograft transplantation: Undersizing grafts can lead to increased rates of clinical and mechanical failure. Knee Surg Sports Traumatol Arthrosc 2019;27:1900-1907. 30767067 10.1007/s00167-019-05398-2

[38] Rosso F Bisicchia S Bonasia DE Amendola A: Meniscal allograft transplantation: A systematic review. Am J Sports Med 2015;43:998-1007. 24928760 10.1177/0363546514536021

[39] Fairbank TJ: Knee joint changes after menisectomy. J Bone Joint Surg Br 1948;30B:664-670. 18894618

[40] Wojdasiewicz P Poniatowski ŁA Szukiewicz D: The role of inflammatory and anti-inflammatory cytokines in the pathogenesis of osteoarthritis. Mediators Inflamm 2014;2014:561459. 24876674 10.1155/2014/561459 PMC4021678

[41] Sadlik B Gobbi A Puszkarz M Klon W Whyte GP: Biologic inlay osteochondral reconstruction: Arthroscopic one-step osteochondral lesion repair in the knee using morselized bone grafting and hyaluronic acid-based scaffold embedded with bone marrow aspirate concentrate. Arthrosc Tech 2017;6:e383. 28580256 10.1016/j.eats.2016.10.023 PMC5443400

